# Automated cardiovascular MR myocardial scar quantification with unsupervised domain adaptation

**DOI:** 10.1186/s41747-024-00497-3

**Published:** 2024-08-14

**Authors:** Richard Crawley, Sina Amirrajab, Didier Lustermans, Robert J. Holtackers, Sven Plein, Mitko Veta, Marcel Breeuwer, Amedeo Chiribiri, Cian M. Scannell

**Affiliations:** 1 School of Biomedical Engineering & Imaging Sciences, King’s College London, London, UK; 2 Department of Biomedical Engineering, Eindhoven University of Technology, Eindhoven, the Netherlands; 3 Cardiovascular Research Institute Maastricht (CARIM), Maastricht University, Maastricht, the Netherlands; 4 Department of Radiology and Nuclear Medicine, Maastricht University Medical Center, Maastricht, the Netherlands; 5 Leeds Institute of Cardiovascular and Metabolic Medicine, University of Leeds, Leeds, UK

**Keywords:** Artificial intelligence, Cardiovascular magnetic resonance, Image processing (computer assisted), Late gadolinium enhancement, Myocardium

## Abstract

**Abstract:**

Quantification of myocardial scar from late gadolinium enhancement (LGE) cardiovascular magnetic resonance (CMR) images can be facilitated by automated artificial intelligence (AI)-based analysis. However, AI models are susceptible to domain shifts in which the model performance is degraded when applied to data with different characteristics than the original training data. In this study, CycleGAN models were trained to translate local hospital data to the appearance of a public LGE CMR dataset. After domain adaptation, an AI scar quantification pipeline including myocardium segmentation, scar segmentation, and computation of scar burden, previously developed on the public dataset, was evaluated on an external test set including 44 patients clinically assessed for ischemic scar. The mean ± standard deviation Dice similarity coefficients between the manual and AI-predicted segmentations in all patients were similar to those previously reported: 0.76 ± 0.05 for myocardium and 0.75 ± 0.32 for scar, 0.41 ± 0.12 for scar in scans with pathological findings. Bland-Altman analysis showed a mean bias in scar burden percentage of -0.62% with limits of agreement from -8.4% to 7.17%. These results show the feasibility of deploying AI models, trained with public data, for LGE CMR quantification on local clinical data using unsupervised CycleGAN-based domain adaptation.

**Relevance statement:**

Our study demonstrated the possibility of using AI models trained from public databases to be applied to patient data acquired at a specific institution with different acquisition settings, without additional manual labor to obtain further training labels.

**Graphical Abstract:**

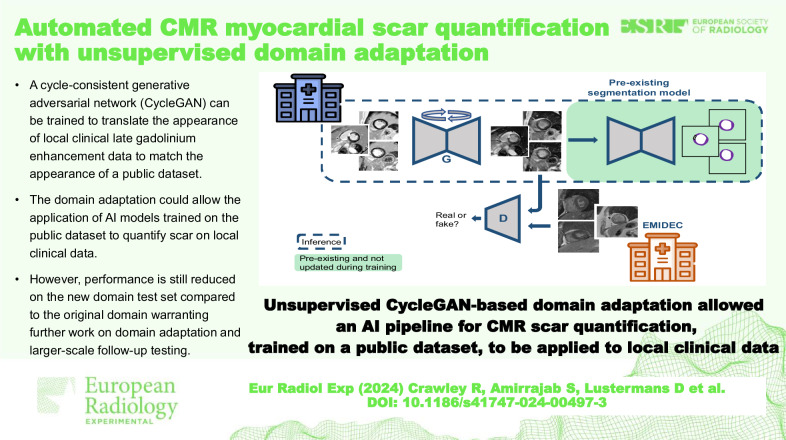

## Background

Late gadolinium enhancement (LGE) cardiovascular magnetic resonance (CMR) is the non-invasive reference standard to assess the presence and extent of myocardial scar [1]. Semiautomated methods for scar quantification have been previously validated as accurate [2] and reproducible [3] but the required manual contouring of the endocardial and epicardial borders remains time-consuming [4]. Therefore, a standard clinical practice still relies on image visual interpretation. Fully automated scar quantification instead could aid clinical translation and allow accurate measurements of the size and transmurality of myocardial infarction and could improve observer confidence.

We have previously developed an automated artificial intelligence (AI)-based CMR scar quantification pipeline [5]. The deep learning models were trained with publicly available data from a single scanner vendor as part of a competition, the “Automatic evaluation of myocardial infarction from delayed-enhancement cardiac magnetic resonance−EMIDEC” challenge [6]. Whilst such competitions are useful to promote research, models trained on selective and uniform acquisition data may not be applicable in real-world clinical applications.

It has been widely reported that acquisition-specific differences represent a “domain shift” in CMR and that this domain shift degrades the performance of deep learning models [7–9]. For LGE images, there are large differences between domains related to the acquisition parameters as well as to the type, concentration, dose, and injection protocol of the gadolinium-based contrast agent, making the application of previously trained models challenging [5]. At our institution, LGE is performed using scanners of varying field strength and from multiple vendors with a dark-blood sequence optimized to null the blood pool signal for improved scar-to-blood contrast [7]. Therefore, domain adaptation steps are required to enable clinical deployment of the EMIDEC model on our local data.

Recent work has demonstrated that cycle-consistent generative adversarial networks (CycleGANs) can be used for unpaired image-to-image translation [10], with some examples including stain normalization of histopathological slides [11]; adapting magnetic resonance imaging (MRI) images from different scanners [12, 13]; and the translation of MRI images to computed tomography [14].

The aim of this study was to develop a CycleGAN-based domain adaptation scheme that allows the use of the previously trained automatic scar quantification pipeline without additional training labels or retraining.

## Methods

### Datasets

The data included a development set to train the CycleGAN and an independent held-out test set to evaluate the automated scar quantification. Written informed consent was obtained from all patients for inclusion in this research. The study was approved by a UK Research Ethics Committee (reference: 15/NS/0030) and complies with the Declaration of Helsinki.

#### Development set

The CycleGAN training required unlabeled, unpaired images from both source and target domains. The source domain images consisted of the original EMIDEC dataset (150 patients), as previously reported [6]. The target domain images consisted of retrospectively included two-dimensional dark-blood phase-sensitive inversion-recovery LGE data from a 3-T Achieva TX scanner (Philips Healthcare, Best, the Netherlands) at King’s College London acquired at least 10 min after an intravenous injection of 0.2 mmol/kg gadobutrol (Gadovist, Bayer, Berlin, Germany), acquisition sequence as previously described [15] with further details in the Supplementary Materials. It included a convenience sample of 150 patients with 1979 slices from previous studies [16, 17].

#### Independent test set

For independent testing, 44 patients undergoing dark-blood LGE examinations, acquired in the same way as the development set, for clinical investigation of coronary artery disease were retrospectively enrolled. The baseline and demographic characteristics of the independent test set population are shown in Supplementary Materials. Manual labels were generated using commercially available software (cvi42, Circle CVI, Calgary, Alberta, Canada). Labeling was performed by a European Association of Cardiovascular Imaging CMR level 3 observer with > 3 years of full-time experience in CMR (R.C.). Labeling involved the manual tracing of the endocardial and epicardial boundaries followed by placing of a region of interest in the remote normal myocardium and identification of the highest signal intensity value within the whole myocardium. Areas of infarcted myocardium were identified as 5 standard deviations (SDs) above the mean signal of remote normal myocardium, as this method was found to be accurate [2] with minimal interobserver and intraobserver variability [18] for dark-blood LGE segmentation.

### Automated scar quantification

The scar quantification method has been previously described [5]. It is a cascaded pipeline of three models: (1) a bounding box regression network to detect the heart; (2) a U-Net model [19] to segment the myocardium; and (3) a U-Net model to segment scar, if present.

### Domain adaptation

**Fig. 1:**
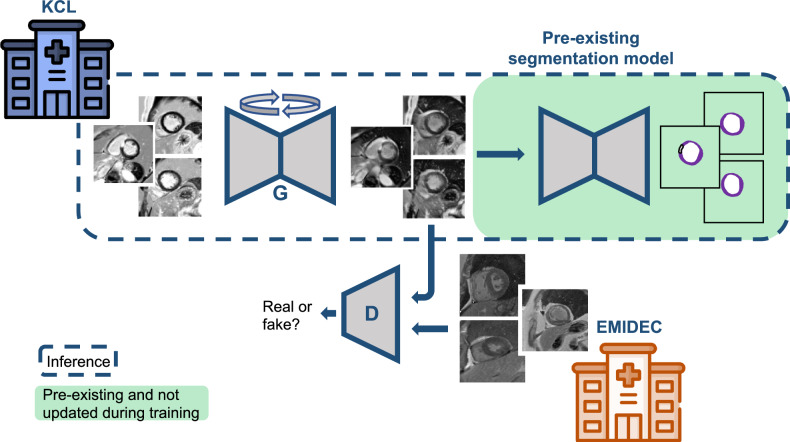
Representation of the CycleGAN-based domain adaptation. Note that the CycleGAN training requires two generators and associated discriminator models (the second is not shown here, for simplicity). Hospital icons created by Freepik–Flaticon (https://www.flaticon.com/authors/freepik)

Figure 1 shows a schematic representation of the domain adaptation scheme. A CycleGAN model was trained to translate local dark-blood clinical data to appear similar to EMIDEC data so that it can be processed with the scar quantification pipeline trained on EMIDEC data. Separate CycleGAN models are used between steps 1 and 2 and between steps 2 and 3 in the scar quantification pipeline to adapt the input to the two U-Net models. CycleGAN-based domain adaptation was not used prior to the first step of the scar quantification method [5] which predicts a region of interest around the LV myocardium.

A CycleGAN used a conditional generator to map the source domain to the target domain. The generator was trained adversarially, with a discriminator attempting to distinguish real and generated images. Simultaneously, a second generator (with associated discriminator) maps data from the target domain back to the source domain. In addition to the GAN loss, cycle consistency was enforced such that an image mapped from the source to the target domain and then back (or vice versa) should match the original input, to preserve structural image information when translating one domain to the other.

The two generators used 2-D U-Net architectures [19] consisting of five resolution steps with two convolutional blocks each, with the number of convolutional filters doubling each step from 32. The training used the Adam optimizer with ${\beta }_{1}=0.5,{\beta }_{2}=0.99$ and learning rate linearly decaying to 0 from 0.0001. The batch size was 2, affine image augmentations were used (as detailed in the Supplementary Materials), and the models were trained for 100 epochs with the best model chosen by visual assessment. The GAN loss as described by de Bel et al [11] was used with the cycle-consistency term weighted by a factor of 6 and the identity term weighted by 0.4, determined after initial experimentation. The training script is provided at https://github.com/q-cardIA/lge-cyclegan.

### Statistical analysis

The performances of the AI scar and myocardium segmentation were assessed using the Dice similarity coefficient (DSC) which quantifies the overlap between AI and manual segmentations between 0 (no overlap) and 1 (complete overlap). Scar quantification methods were compared with a nonparametric test, *i.e.*, the Wilcoxon signed-rank test. Bland-Altman analysis of the derived scar burdens was performed to assess agreement.

## Results

**Fig. 2:**
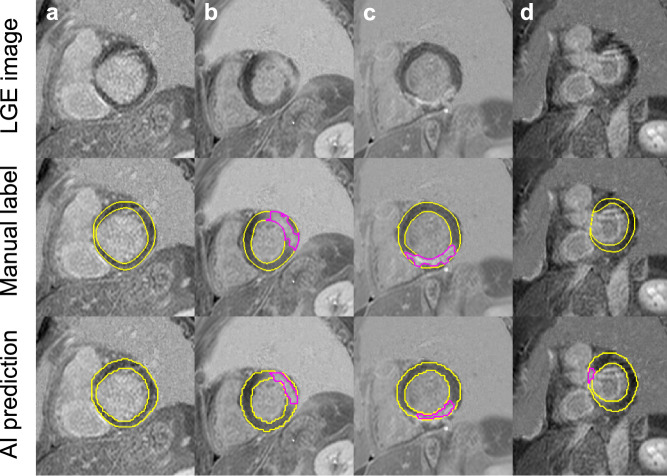
A single image slice from four patients showing representative performance, comparing artificial intelligence (AI) predictions to manual myocardium (yellow) and scar (pink) contours. Column **a**: well-matched myocardium contours in a case without scar. Columns **b** and **c**: good, albeit imperfect overlap for both myocardium and scar in cases with infarction. Column **d**: false positive scar prediction by AI in a slice with visible left ventricular outflow tract

**Fig. 3:**
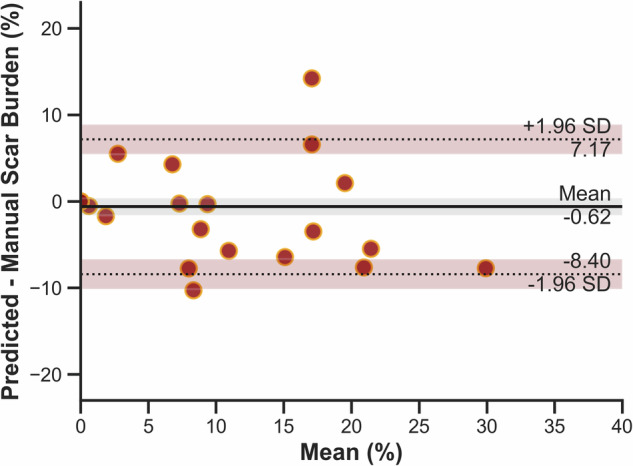
Bland-Altman analysis showing a mean bias in percentage scar burden between the artificial intelligence-predicted and manual quantification of -0.62% with limits of agreement from -8.40% to 7.17%. The dotted lines represent the limits of agreement, and the shaded regions are 95% confidence intervals around each of them

Representative slices from the test set are visualized in Fig. 2, comparing the myocardium and scar segmentations of the AI, with domain adaptation, *versus* the manual observer. The mean (± SD) DSC value between the manual and automatic segmentations was 0.76 ± 0.05 for the myocardium and 0.75 ± 0.32 for the scar. The overall scar segmentation DSC was biased by the perfect overlap in patients without scar and the scar DSC in the subset of patients that were positive for scar was 0.41 ± 0.12. There was no statistically significant difference between the manual scar analysis and the automated AI scar burden quantification (*p* = 0.170). Figure 3 shows the Bland-Altman analysis comparing the scar burden. Total run time was lower than 3 min per patient including data loading, processing, and all model inference.

## Discussion

In this study, we developed an unsupervised domain adaptation scheme using CycleGAN models to translate the image appearance of a single vendor publicly available LGE CMR data to match local clinical data acquired using a different scanner vendor with different acquisition parameters. We demonstrated the possibility of using AI models trained from public databases to be applied to patient data acquired at a specific institution with different acquisition settings. Of note, this approach did not require additional manual labor to obtain further training labels and could help to exploit the growing amounts of publicly available data.

Previous studies proposing automated myocardial scar quantification have trained and tested their models using a single cohort from a single study at one field strength with similar acquisition parameters and no external validation [20]. The performance reported in our study is slightly lower than those previously published [20], but this is expected given our unsupervised domain adaptation approach with the external test dataset. The mean (± SD) DSC value between the manual and automatic segmentations was 0.76 ± 0.05 for the myocardium and 0.75 ± 0.32 for the scar. The higher variability of scar DSC is due to the relatively small size of scar regions to be segmented which means that even small misalignments of the AI segmentation can lead to low DSC values [21] and because patients with no scar correctly predicted as having no scar achieve a perfect DSC of 1, so there is a wide range of scar DSC values. Nevertheless, the automated scar burden showed reasonable agreement with manual quantification (Fig. 3), and this agreement would be expected to increase if the pipeline were to be re-trained with data from the target domain.

Due to its time-intensive nature, myocardial scar quantification is not routinely performed in clinical practice. Furthermore, the lack of availability of labeled data has resulted in scar quantification rarely being studied with automated AI algorithms. An automated algorithm could lead to increased routine use, leading to a more reproducible and accurate assessment tool. In addition, automated AI-based quantification of scars could be combined with automated quantitative myocardial perfusion assessment [22].

In conclusion, this study demonstrates that unsupervised domain adaptation using a CycleGAN model may facilitate the clinical deployment of AI models across centers. Our application for LGE imaging in CMR could allow automated scar quantification of dark-blood local clinical data using a model previously trained on public data. It is acknowledged that this is a small retrospective evaluation, only considering ischemic scar and the clinical utility of the scar quantification tool will need to be assessed prospectively at a larger scale with scars of different etiology.

### Supplementary information

**Additional file 1: Supplementary Table 1:** Patient demographics (N = 44), values are n (%) or mean ± SD. Left ventricular end-diastolic volume is indexed to body surface area calculation. **Supplementary Table 2:** The parameters used for the data augmentation in the bounding box training. U(a, b) denotes that the parameter value was randomly sampled from a uniform distribution on the interval [a, b]. Translation and scaling were applied independently in x and y. Parameters for translation and scaling are given as a proportion of the image size.

## Data Availability

The code for model training is provided at https://github.com/q-cardIA/lge-cyclegan. The EMIDEC challenge data is available at https://emidec.com/. Local clinical data is not made publicly available due to institutional restrictions.

## Abbreviations

AI: Artificial intelligence
CMR: Cardiovascular magnetic resonance
CycleGAN: Cycle-consistent generative adversarial network
DSC: Dice similarity coefficient
EMIDEC: Automatic evaluation of myocardial infarction from delayed-enhancement cardiac magnetic resonance
LGE: Late gadolinium enhancement
MRI: Magnetic resonance imaging
SD: Standard deviation
