# Supplementary material for: Automated cardiovascular MR myocardial scar quantification with unsupervised domain adaptation
Source: Eur Radiol Exp. 2024 Aug 14;8:93. doi: 10.1186/s41747-024-00497-3 (PMC11324636; doi:10.1186/s41747-024-00497-3)
Supplement: Supplementary file 1 — Additional file 1: Supplementary Table 1: Patient demographics (N = 44), values are n (%) or mean ± SD. Left ventricular end-diastolic volume is indexed to body surface area calculation. Supplementary Table 2: The parameters used for the data augmentation in the bounding box training. U(a, b) denotes that the parameter value was randomly sampled from a uniform distribution on the interval [a, b]. Translation and scaling were applied independently in x and y. Parameters for translation and scaling are given as a proportion of the image size. [file 41747_2024_497_MOESM1_ESM.pdf]

Automated Cardiovascular MR myocardial scar quantification with unsupervised domain adaptation
ELECTRONIC SUPPLEMENTARY MATERIAL

Dark-blood LGE acquisition

Dark-blood LGE imaging used an ECG-triggered segmented spoiled gradient-echo PSIR sequence acquired during a breath-hold on a clinical 3 Tesla system (Achieva TX, Philips Healthcare, Best, the Netherlands) equipped with a 32-channel cardiac phased-array coil. Images were acquired >10 minutes after an intravenous injection of 0.2 mmol/kg gadobutrol (Gadovist, Bayer, Berlin, Germany). Typical sequence parameters were: TR 3.5 ms, TE 2.0 ms, flip angle 25°, PSIR reference readout flip angle 5°, acquired resolution 1.61 × 1.61 mm2, reconstructed resolution 0.65 × 0.65 mm2, slice thickness 8 mm. Images are acquired with an inversion time (TI) that nulls blood signal (instead of nulling normal myocardium), as described in (1).

Patient baseline and demographic characteristics

Age (years)	63 ± 12
Male	29 (66)
LV end-diastolic volume (ml/m^2)	83 ± 23
LV ejection fraction (%)	54 ± 13
LV ejection fraction < 50%	11 (25)
Maximum wall thickness (mm)	10 ± 2
Ischemic LGE presence	17 (39)
LGE > 15%	9 (21)

Table 1: *Patient demographics (N = 44), values are n (%) or mean ± SD. Left ventricular end-diastolic volume is indexed to body surface area calculation.*

Data augmentation

**Type of Augmentation**	**Value**
Translation	$\mathcal{U}$(-0.15, 0.15)
Scaling	$\mathcal{U}$(0.85, 1.15)
Rotation (degrees)	$\mathcal{U}$(-90, 90)

Table 2: *The parameters used for the data augmentation in the bounding box training. U(a, b) denotes that the parameter value was randomly sampled from a uniform distribution on the interval [a, b]. Translation and scaling were applied independently in x and y. Parameters for translation and scaling are given as a proportion of the image size.*

References

1. Holtackers RJ, Chiribiri A, Schneider T, Higgins DM, Botnar RM. Dark-blood late gadolinium enhancement without additional magnetization preparation. Journal of Cardiovascular Magnetic Resonance. 2017;19(1):64. doi: 10.1186/s12968-017-0372-4.
